# Proceedings of the North American Society of Head and Neck Pathology, Los Angeles, CA, March 20, 2022: SWI/SNF-deficient Sinonasal Neoplasms: An Overview

**DOI:** 10.1007/s12105-022-01416-x

**Published:** 2022-03-21

**Authors:** Abbas Agaimy

**Affiliations:** Institute of Pathology, Friedrich-Alexander-University Erlangen-Nürnberg, University Hospital, Erlangen, Germany

**Keywords:** SWI/SNF complex, SMARCB1, Teratocarcinosarcoma, Head and neck, Epithelioid sarcoma, Rhabdoid tumor

## Abstract

The pathology of poorly differentiated sinonasal malignancies has been the subject of extensive studies during the last decade, which resulted into significant developments in the definitions and histo-/pathogenetic classification of several entities included in the historical spectrum of “sinonasal undifferentiated carcinomas (SNUC)” and poorly differentiated unclassified carcinomas. In particular, genetic defects leading to inactivation of different protein subunits in the SWI/SNF chromatin remodeling complex have continuously emerged as the major (frequently the only) genetic player driving different types of sinonasal carcinomas. The latter display distinctive demographic, phenotypic and genotypic characteristics. To date, four different SWI/SNF-driven sinonasal tumor types have been recognized: SMARCB1(INI1)-deficient carcinoma (showing frequently non-descript basaloid, and less frequently eosinophilic, oncocytoid or rhabdoid undifferentiated morphology), SMARCB1-deficient adenocarcinomas (showing variable gland formation or yolk sac-like morphology), SMARCA4-deficient carcinoma (lacking any differentiation markers and variably overlapping with large cell neuroendocrine carcinoma and SNUC), and lastly, SMARCA4-deficient sinonasal teratocarcinosarcoma. These different tumor types display highly variable immunophenotypes with SMARCB1-deficient carcinomas showing variable squamous immunophenotype, while their SMARCA4-related counterparts lack such features altogether. While sharing same genetic defect, convincing evidence is still lacking that SMARCA4-deficient carcinoma and SMARCA4-deficient teratocracinosarcoma might belong to the spectrum of same entity. Available molecular studies revealed no additional drivers in these entities, confirming the central role of SWI/SNF deficiency as the sole driver genetic event in these aggressive malignancies. Notably, all studied cases lacked oncogenic *IDH2* mutations characteristic of genuine SNUC. Identification and precise classification of these entities and separating them from SNUC, NUT carcinoma and other poorly differentiated neoplasms of epithelial melanocytic, hematolymphoid or mesenchymal origin is mandatory for appropriate prognostication and tailored therapies. Moreover, drugs targeting the SWI/SNF vulnerabilities are emerging in clinical trials.

## Introduction

The chromatin remodeling Switch/ Sucrose non-fermentable (SWI/SNF) complex is a highly conserved complex of >20 genes mapped to different chromosomal regions [1]. While the precise function of this complex is still largely unknown, its role in remodeling the chromatin, and hence, regulating the processes of gene expression and cell differentiation has been widely appreciated [1]. In general, the different SWI/SNF complex components function as tumor suppressors [1]. Accordingly, it is inactivating mutations leading to loss of protein function and gene deletions that are responsible for tumor initiation in the SWI/SNF-driven entities [1]. Additional potential mechanisms of SWI/SNF inactivation include epigenetic mechanisms as well. With few exceptions, SWI/SNF inactivating gene abnormalities lead to universal loss of the respective protein of the subunit affected by the genetic alternation, irrespective of the type of gene defect. This, together with the ever-emerging commercially available specific antibodies against different SWI/SNF proteins, made the SWI/SNF immunohistochemistry a powerful tool in identifying and classifying SWI/SNF-driven neoplasms [2–5].

Remarkably, there seems to be a striking organ-specific clustering of mutations in certain SWI/SNF genes, so that *ARID1A* mutations prevail in genital tract malignancies while *SMARCB1* and *SMARCA4* mutations predominate among SWI/SNF-driven neoplasms of soft tissue and lung, respectively [5]. In the sinonasal tract, to date only SMARCB1 (INI1) and SMARCA4 have been recurrently associated with specific tumor types. This highly selective phenomenon might reflect the diverse SWI/SNF assembly variants found in the stem cells and their progenitors in different organs [6]. This review summarizes the pertinent clinicopathological features of those recently described SWI/SNF-related sinonasal neoplasms that are planned to be included in the upcoming WHO classification.

### SMARCB1-Deficient Sinonasal Carcinoma

*SMARCB1*, mapped to chromosomal region 22q11.2, is a core subunit of the SWI/SNF complex [2]. Like other tumor suppressor proteins, SMARCB1 is globally expressed in the nuclei of all human cell types [2–5]. Loss of SMARCB1 because of biallelic inactivation of its gene in a subset of poorly or undifferentiated sinonasal carcinoma has been first described in 2014 by two independent groups, followed by a few additional series including one large multi-institutional series (n=39) [7, 8]. To date, <150 cases have been reported [7–12].

These tumors show a slight predominance of males with a mean age of 52 years (range, 19 to 89). They present as locally advanced disease (mostly T4). The paranasal sinuses, mainly the ethmoids, are involved in majority of cases with variable involvement of the nasal cavity. The disease follows an aggressive clinical course resulting in death of 56% of patients at a median interval of 16 months [10]. Only one third of patients were alive without disease at last follow-up [10].

**Fig. 1:**
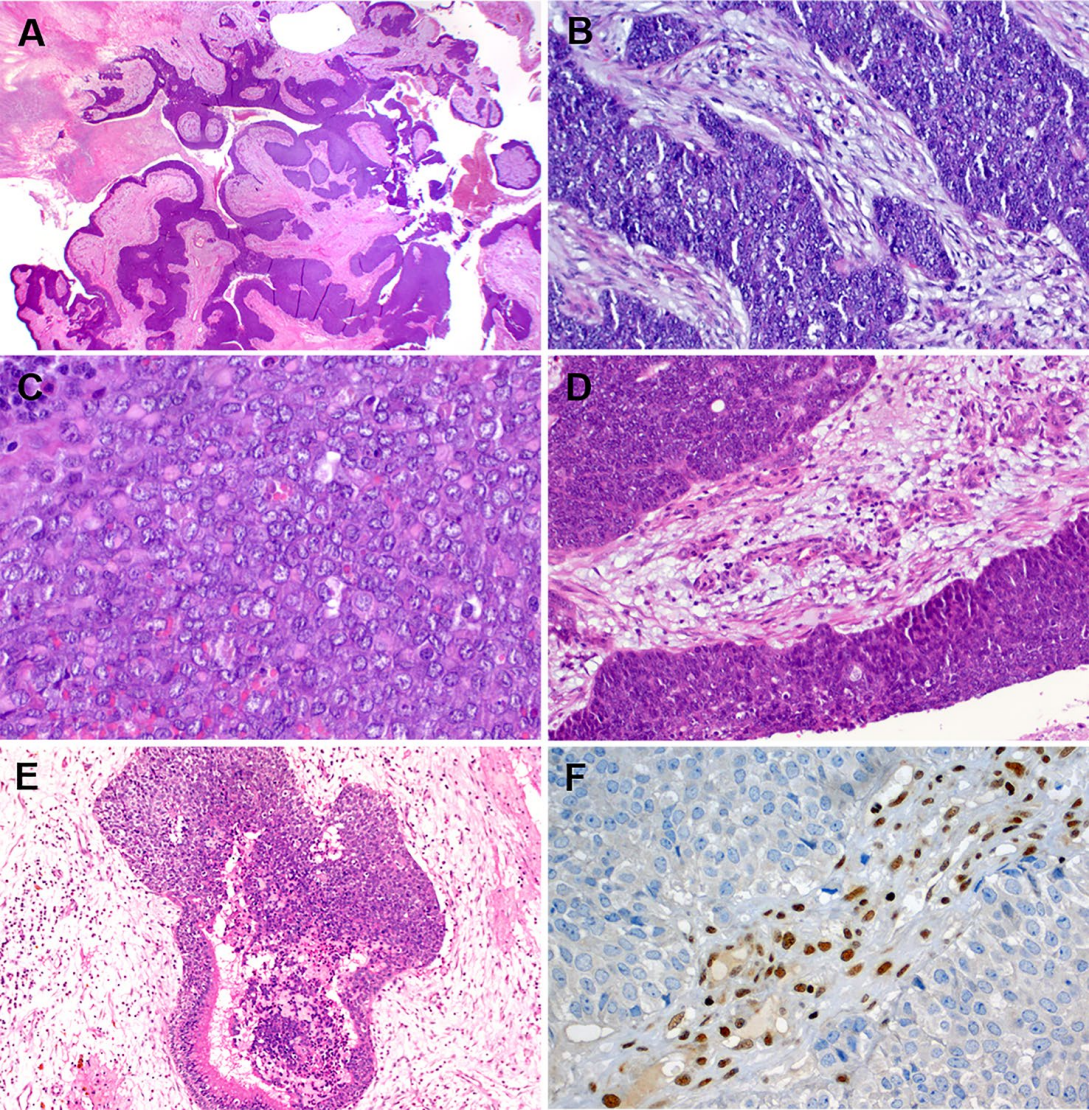
Representative examples of SMARCB1-deficient sinonasal carcinoma. **A** basaloid (dark-stained) monomorphic cells replacing the surface epithelium and showing extensive downgrowth along preexisting glands resulting in combined exophytic-endophytic papillary pattern at low-power. **B** monomorphic basaloid cell morphology with prominent stromal desmoplasia. **C** numerous rhabdoid cells are interspersed among undifferentiated basaloid cells. **D** replacement of the surface mucosa by tumor tissue mimicking carcinoma in situ. **E** partial downgrowth along deeper respiratory glands. **F** complete loss of SMARCB1 is definitional for these tumors

**Fig. 2:**
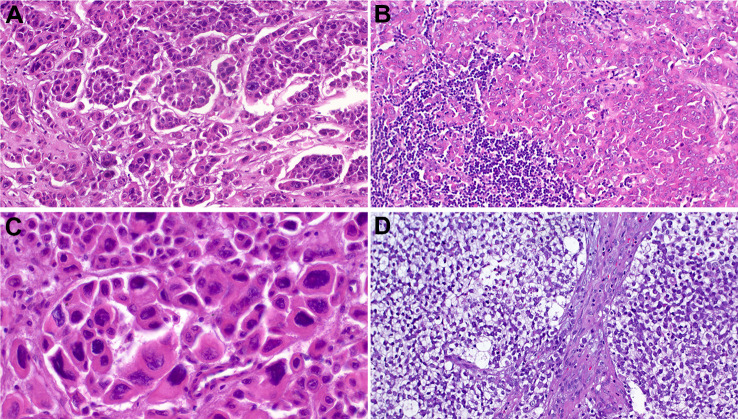
Representative examples of uncommon features in SMARCB1-deficient sinonasal carcinoma. **A** eosinophilic (pinkish) cells forming prominent variably sized cellular nests. **B** lymph node metastasis of an eosinophilic tumor showing compact nests, superficially reminiscent of nonkeratinizing squamous cell carcinoma or apocrine salivary duct carcinoma. Rarely, smudgy bizarre looking cells (**C**) and clear cells (**D**) are seen

Like most other SMARCB1-deficient neoplasms, the tumors display a monomorphic cytology lacking significant pleomorphisms or bizarre-looking nuclei. Majority of cases (60%) show a predominance of basaloid morphology, occasionally indistinguishable from non-keratinizing poorly differentiated basaloid squamous cell carcinoma (SCC). However, frankly squamous cell features and keratinization are absent. A smaller fraction of cases (30%) displays a “pinkish” eosinophilic, plasmacytoid/rhabdoid cell morphology [7–12]. Scattered plasmacytoid and rhabdoid cells can be identified, also in the basaloid tumors, upon scrutiny [25]. Although focal glandular differentiation was mentioned in a few cases in the initial studies, tumors featuring prominent gland formation have been later redefined as adenocarcinomas (see section on adenocarcinoma below). A few cases contain limited foci of clear cells. Spindle cell “sarcomatoid” pattern is rare. No conventional surface dysplasia or carcinoma in situ have been observed, but a variable degree of surface pagetoid spread (occasionally mimicking in situ carcinoma) may be seen [7, 8, 10]. Representative examples of the common and variant features of these tumors are depicted in Figs. 1 and 2.

By immunohistochemistry, SMARCB1-deficient sinonasal carcinomas express uniformly pankeratins (97%) and variably CK5 (64%), p63 (55%) and CK7 (48%) [10]. They are negative for high-risk Human Papillomavirus, Epstein-Barr virus, and NUT [7, 8, 10]. Like SWI/SNF-deficient neoplasms in different organs, SMARCB1-deficient sinonasal carcinoma may show focal immunoreactivity for different neuroendocrine markers [10, 13]. SMARCB1 loss is definitional. Rare co-loss of SMARCA2 but not SMARCA4 or ARID1A may be observed [10]. Biallelic (homozygous) or monoallelic (heterozygous) deletions affecting the *SMARCB1* gene locus are detectable by FISH in majority of cases [8, 10]. Although no detailed prospective studies on histology-tailored therapy for this tumor type are available, an exaggerated response to platinum-based chemotherapy has been observed in anecdotal cases, indicating the need to recognize these tumors correctly, so that their most suitable therapeutic strategies can be assessed reliably in the future [7, 10]. Cases tested with larger gene panels revealed no additional driver mutations, in particular, *IDH2* mutations were absent underlining their distinctness from SNUC [14, 15].

#### Differential Diagnosis

Based on histological pattern, SMARCB1-deficient carcinoma has been previously likely included in the historical spectrum of SNUC, basaloid nonkeratinizing SCC or myoepithelial carcinoma. Its distinction from other epithelial and mesenchymal basaloid neoplasms is based on combined sets of clinicopathological and genotypic features (Table 1). Rare myoepithelial carcinomas with rhabdoid features may display SMARCB1 loss and, when presenting in the sinonasal tact, may mimic SMARCB1-deficient sinonasal carcinomas [16]. The latter however do not express myoepithelial markers such as S100, SOX10, calponin, smooth muscle actin and others.

**Table 1:** Compared clinicopathologic features and genotypes of SWI/SNF-deficient sinonasal maliganancies and their main differential diagnoses

Entity	Papillary growth	Cell type	Keratinization	Surface dysplasia	Squamous markers	Bizarre looking cells	p16	EBV	Defining IHC marker	Genotype
SMARCB1-deficient carcinoma	Occasional	Basaloid or eosinophilic, rhabdoid	Absent	Absent*	Heterogeneous	Absent	Usually neg	neg	SMARCB1 loss	*SMARCB1* mutations or deletions
SMARCB1-deficient adenocarcinoma	Absent	Mainly eosinophilic, glands or yolk sac-like	Absent	Absent*	Heterogeneous	Absent	Usually neg	neg	SMARCB1 loss	*SMARCB1* mutations or deletions
SMARCA4-deficient carcinoma	Rare or absent	Large cells, rarely basaloid	Absent	Absent	Absent	Absent	neg	neg	SMARCA4 loss	Loss-of-function/ truncating mutations in *SMARCA4*
TCS	Rare or absent	Diversity of cell and tissue types	Absent/rare	Absent	Limited to squamous foci	Absent	neg	neg	Morphology, SMARCA4 loss	Loss-of-function/ truncating mutations in *SMARCA4*, *CTNNB1* mutations
Conventional basaloid SCC	Uncommon	Basaloid	May be present	Present	Diffuse	May be present	neg	neg	None (exclusion of others)	No specific, *TP53* mutations
HPV-realted SCC	Frequent	Basaloid	Rare or absent	May be present	Diffuse	May be present	Diffuse	neg	p16	HR-HPV
HPV-related multiphenotypic carcinoma	Uncommon	Variable	Uncommon	Present	Variable	Frequent	Diffuse	neg	Based on histology pattern (various salivary-types)	HR-HPV (mainly 33 and other rare types)
Lymphoepithelial carcinoma	Rare or absent	Large cells	Absent	Absent	Diffuse	Usually absent	neg	pos	EBV-ISH, TP53 diffuse	EBV
Adamantinoma-like Ewing sarcoma	Rare or absent	Basaloid small cells	May be present**	Absent	Usually diffuse	Absent	neg	neg	CD99, NKX3.2***	*EWSR1* fusions
Solid aRMS	Absent	Basaloid small cells	Absent	Absent	Absent	Usually absent	neg	neg	Desmin, myogenin	*FOXO1-PAX* fusions
NUT carcinoma	Rare or absent	Basaloid small or large cells	May be present**	Absent	Usually diffuse	Absent	Usually neg	neg	NUT IHC diffuse	*NUTM1* fusions
Poorly differentiated NEC	Absent	Small or large cells	Absent	Absent	neg	Usually absent	May be diffuse	neg	NE cytology, Synaptophysin, Chromogranin****	*Rb1* & *TP53* mutations, may be HR-HPV +
SNUC	Rare or absent	Large cells	Absent	Absent	Absent	Absent	neg/+	neg	Lack of squamous & glandular markers, IDH IHC+	*IDH2* mutations

* pagetoid spreading may mimic surface dysplasia. **if present usually abrupt. *** note that CD99 is not specific to Ewing. **** patchy NE expression is frequent in SWI/SNF and other entities and should not be confused with NEC

*aRMS* alveolar rhabdoomyosarcoma, *IHC *immunohistochemistry, *NEC* neuroendocrine carcinoma, *HR-HPV *high-risk Human Papillomavirus, *SCC *squamous cell carcinoma, *SNUC *sinonasal undifferentiated carcinoma, *TCS *teratocarcinosarcoma

### SMARCB1-deficient Sinonasal Adenocarcinoma

This rare variant in the spectrum of SMARCB1-deficient sinonasal carcinoma is defined by presence of unequivocal gland formation [17]. To date, <20 cases have been described [17, 18]. A higher predilection for males (5:1) is observed compared to non-glandular SMARCB1-deficient sinonasal carcinomas. Both undifferentiated and gland-forming tumor types present at a comparable median age (52 versus 57 years; range, 19 to 89 versus 21 to 82, respectively). The nasal cavity represents the major site affected with some tumors involving multiple sinonasal sites. Selective involvement of the ethmoid or the maxillary sinuses is less common.

Histologically, these tumors feature a predominance of oncocytoid/plasmacytoid cell morphology with variable, but unequivocal gland formation characterized by presence of tubules, cribriforming, intracellular and/or luminal mucin and foci with yolk sac tumor-like features [17, 18]. The latter may closely resemble the pattern of secretory endometrium seen in gonadal yolk sac tumors. The sieve-like reticular/ microcystic yolk sac pattern may be predominant and rarely represent the sole pattern seen throughout the tumor [18–20]. This variant can be diagnostically challenging if one is not aware of it. The neoplastic cells usually display uniformly high-grade cytology with brisk mitotic activity and frequent foci of necrosis, but rare cases may show a deceptively bland-looking histology.

**Fig. 3:**
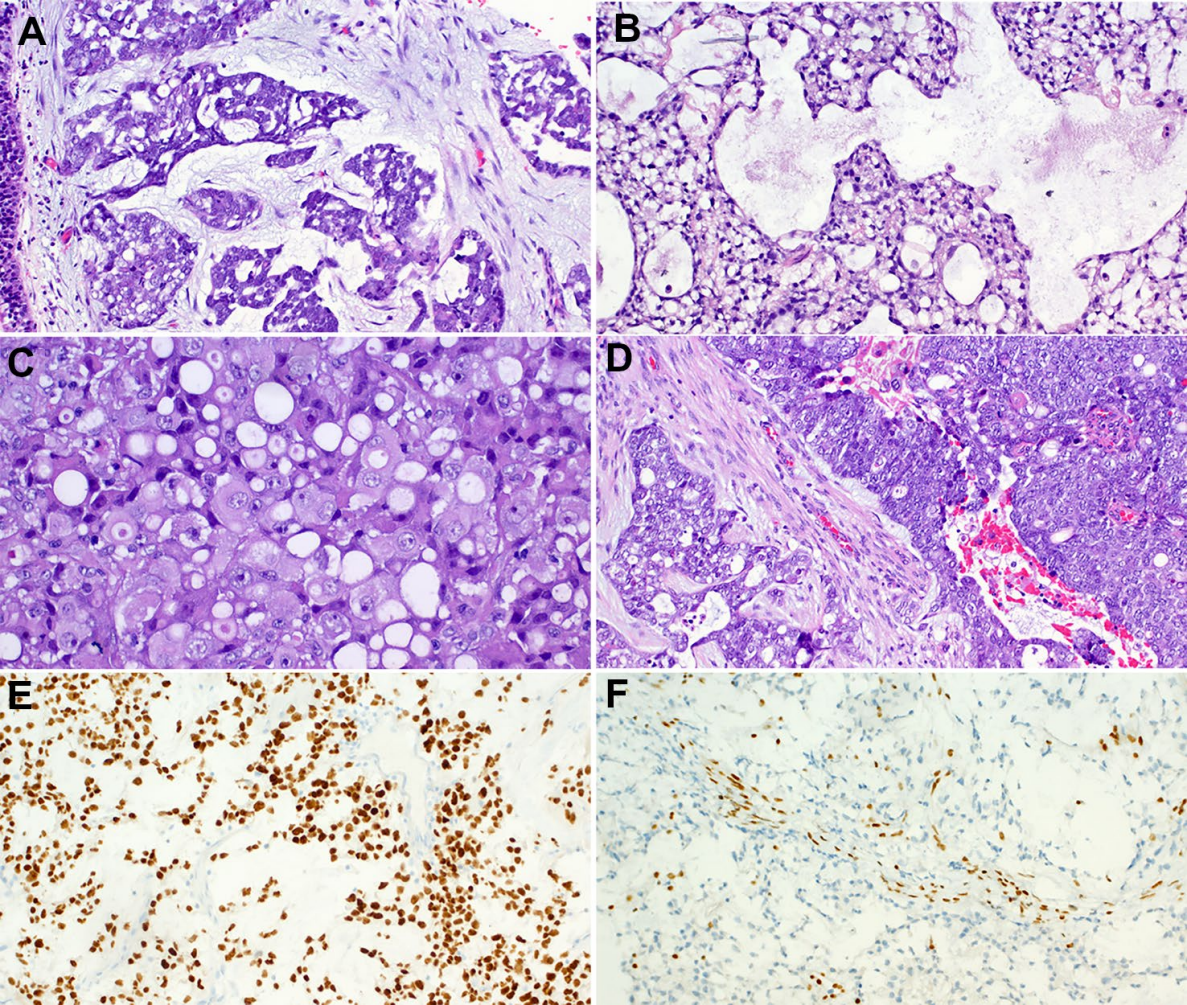
Representative examples of SMARCB1-deficient sinonasal adenocarcinoma. **A** variable admixture of glands, sieve-like pattern or pseudocribriform structures embedded within abundant mucoid stroma, note uniformly high-grade cytology. **B** some tumors display pure yolk sac-like morphology with bland-looking cells. **C** this predominantly solid tumor shows numerous mini-lumina and large eosinophilic cells, highly reminiscent of apocrine salivary duct carcinoma and high-grade non-intestinal adenocarcinoma. **D** transition from adenocarcinoma (left) to solid undifferentiated component (right) may be seen. **E** strong diffuse reactivity with SALL4 combined with pure yolk sac-like pattern, as in this case, represents a major pitfall. **F** complete loss of SMARCB1 is limited to the neoplastic cells

In contrast to the non-glandular SMARCB1-deficient carcinomas, this variant displays more extensive and frequent expression of CK7 (83%) than p40 (33%), [17]. Variable reactivity is observed for glypican-3 (90%), SALL4 (50%), HepPar-1 (50%), CDX2 (27%) CK20 (25%), PLAP (11%) and AFP (10%), irrespective of present or absent yolk sac-like features [17]. Representative examples of the common and variant features of these tumors are depicted in Fig. 3.

#### Differential Diagnosis

SMARCB1-deficient sinonasal adenocarcinoma needs to be distinguished from several mimics including mainly high-grade intestinal and non-intestinal adenocarcinoma, as well as myoepithelial carcinoma, primary and metastatic yolk sac tumor, and metastatic hepatoid adenocarcinoma from the digestive system and other sites.

### SMARCA4-deficient Sinonasal Carcinoma

The catalytic SWI/SNF chromatin remodeling complex subunit SMARCA4 (BRG1) is mapped to chromosomal region 19p13.2 [1]. Like SMARCB1, it is also involved in the regulation of gene transcription, and it hence regulates cell differentiation. Inactivating (either sporadic or germline) *SMARCA4* mutations have emerged as a defining marker for a plethora of highly aggressive malignancies unified by large undifferentiated epithelioid or rhabdoid cells and occurring across different organs (ovary, uterus, mediastinum, lung, digestive tract, and others) [21–27].

Initially reported as rare alternative mechanism in a rare subset of SMARCB1-proficient sinonasal carcinomas [28], SMARCA4 inactivation has latter been recognized as a defining marker for a distinctive subset of aggressive sinonasal epithelial neoplasms characterized by similar morphology as SMARCA4-deficient malignancies in other organs. Uniformity of cytology and keratin expression justified classifying these rare malignancies in the sinonasal tract as carcinomas and not sarcomas. Indeed, the prototypical SMARCA4-deficient thoracomediastinal malignancies (initially considered sarcomas [22]) have been redefined as undifferentiated carcinoma type based on several demographic and clinicopathological characteristics [29]. To date, a total of 22 cases have been reported [28, 30–33]. SMARCA4-deficient sinonasal carcinomas represented 4% of poorly differentiated/undifferentiated epithelial-derived sinonasal malignancies [33], 9% of all SNUCs, and 20% of *IDH2*-wildtype SNUC [30]. Their demographic and clinical features are similar to their SMARCB1-deficient counterparts, but with a striking predilection for men and a lower median age (35–44 vs. 57 years; range, 20–70) [32, 33]. Most tumors originate in the nasal cavity or involve multiple sinonasal sites. In contrast to SMARCB1-deficient carcinomas, and because of their cell morphology and immunophenotype, most SMARCA4-deficient tumors have been initially misclassified as small or large cell neuroendocrine carcinomas and, less frequently, as SNUC or teratocarcinosarcoma [32, 33].

Histologically, sheets of large epithelioid anaplastic cells predominate with variable nesting or trabecular pattern mimicking SNUC. This pattern recapitulates the pattern seen in SMARCA4-deficient undifferentiated malignancies of the thorax, ovary, and other visceral organs [21–24]. Contrasting with their SMARCB1-deficient counterparts, the basaloid small cell pattern is much infrequent in SMARCA4-deficient sinonasal carcinomas. Rhabdoid cells are uncommon but may rarely predominate. Brisk mitotic activity and extensive foci of necrosis are commonly seen. SMARCA4-deficient sinonasal carcinomas lack differentiated features, both histologically (absence of glands, squamous cells, and others) and immunohistochemically (lack of squamous cell marker expression) [32, 33].

**Fig. 4:**
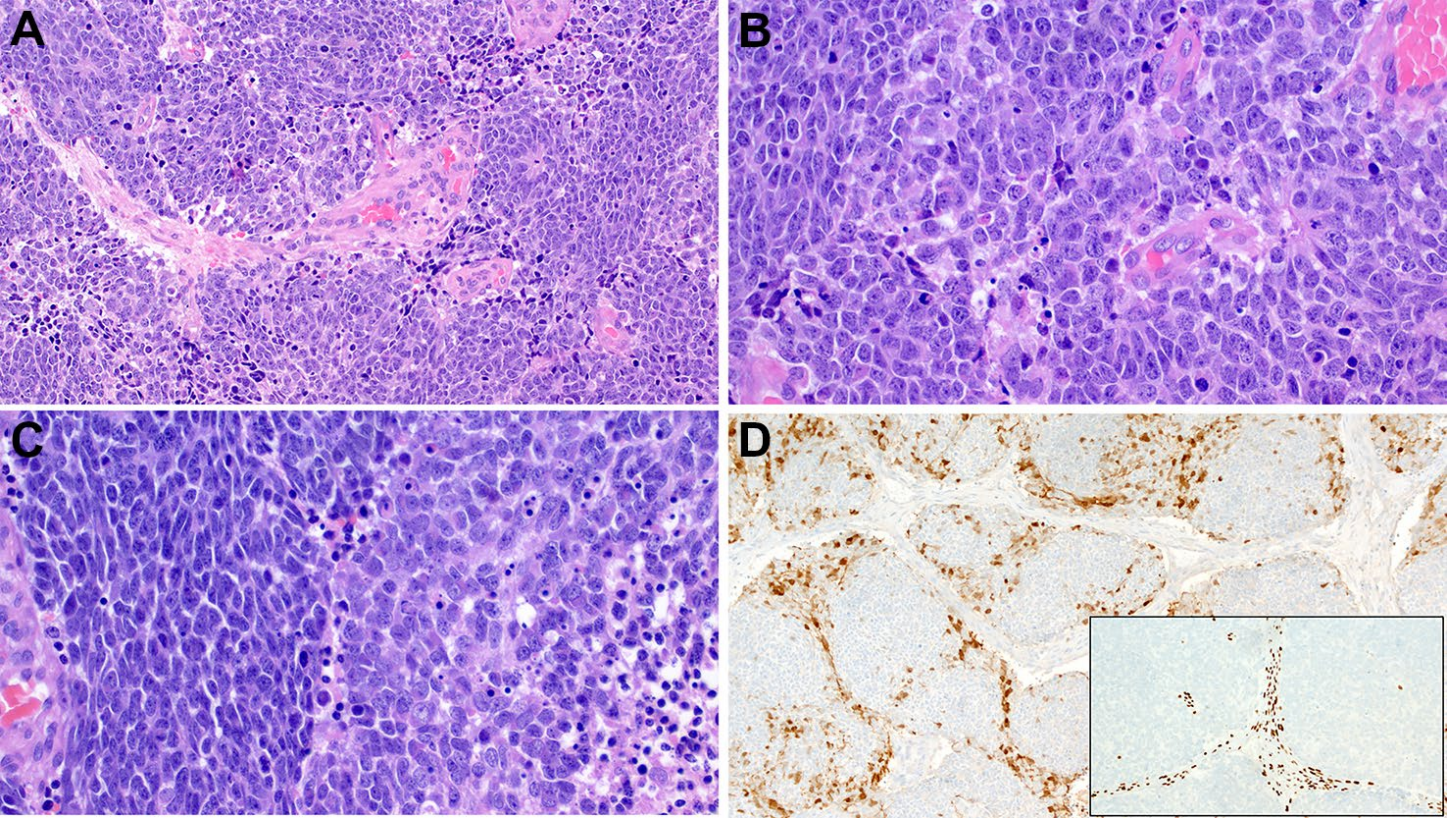
Representative examples of SMARCA4-deficient sinonasal carcinoma. **A** SMARCA4-deficient sinonasal carcinoma show a predominance of large undifferentiated cells with variable lobular pattern mimicking SNUC and large cell neuroendocrine carcinoma. **B** cytological uniformity with variably prominent nuclei and brisk mitotic activity. **C** rarely, abrupt transition from large cell (right) to blue-stained ovoid cells mimicking small cell carcinoma (left) is seen; note foci of necrosis on right. **D** main image: calretinin as well as synaptophysin (not shown) are expressed at the periphery of the cell aggregates suggesting hybrid olfactory-like features. **D** subimage: complete loss of SMARCA4 is the defining feature of these tumors

Abortive neuroendocrine-like rosettes are seen in rare cases. Immunohistochemistry confirms lack of differentiation (consistently positive for pankeratin and rarely CK7 but negative with CK5, p63, p40, p16 and NUT). Notably, variable (mostly focal and weak) reactivity with the neuroendocrine markers is seen including synaptophysin (90%), chromogranin-A (40%) and CD56 (60%) [32]. Rare cases display olfactory-like immunophenotypic features at the periphery of the neoplastic lobules suggesting abortive hybrid differentiation [32]. Global loss of SMARCA4, restricted to the neoplastic cells, is definitional in all cases. Rare cases may show co-loss of SMARCA2 [32]. Retained expression of SMARCB1/INI1 is seen in all cases. Representative examples of the common and variant features of these tumors are depicted in Fig. 4.

SMARCA4-deficient sinonasal carcinoma behaves more aggressively compared to the SMARCB1-deficient tumors; two thirds of patients with follow-up died of their disease within one year [32, 33].

#### Differential Diagnosis

NUT carcinoma (mainly the undifferentiated large or small cell pattern), neuroendocrine carcinomas (including small and large cell types) and Hyams grade 4 olfactory neuroblastoma are the major considerations [34, 35]. Lack of p40 expression is observed in a small subset of NUT carcinoma and is thus not reliable to rule out NUT carcinoma, making the NUT immunohistochemistry mandatory in this differential. In most cases, SMARCA4-deficient sinonasal carcinoma lacks the classical nuclear and chromatin characteristics of true neuroendocrine carcinomas, but they display frequently focal or diffuse but usually weak and heterogeneous expression of the neuroendocrine markers [32]. Diffuse expression of keratins, lack of classical neuroendocrine features, and absence of uniform reactivity for neuroendocrine markers exclude the possibility of olfactory neuroblastoma. As pointed out above, a rare subset of tumors tends to display olfactory-type differentiation limited to the periphery of the neoplastic nests and lobules with coexpression of neuroendocrine markers and calretinin. In contrast to genuine SNUC, all cases tested to date have been negative for *IDH*2 mutations [15, 30, 32, 33].

### SMARCA4-deficient Sinonasal Teratocarcinosarcoma

Teratocarcinosarcoma (TCS) is a rare and highly aggressive malignancy that is virtually confined to the sinonasal tract and has not been reported to occur in other organs. It is defined by a triphasic growth combining teratoma-like, carcinoma-like, and sarcomatous stromal/mesenchymal elements with highly varying neuroectodermal-like components [36]. Several histogenetic theories have been raised to explain their varied multiphenotypic histology including a “germ cell origin”, hence the original name “malignant teratoma” [36]. However, recent developments have shed more light on the molecular pathogenesis and histogenesis of different multiphenotypic tumors, historically considered variants of primitive germ cell neoplasms and referred to by the name “malignant teratoma”. Notably, so-called malignant teratoma of the thyroid has been redefined recently as DICER1-associated somatic malignancy of adults, unrelated to genuine germ cell neoplasia [37, 38]. Likewise, identification of inactivating biallelic *SMARCA4* mutations in the vast majority of sinonasal TCS confirmed the notion that these malignancies are of epithelial somatic origin and are not related to germ cell lineage too [39].

Sinonasal TCSs are exceptionally rare. To date, ~ 130 cases have been reported: mostly as single case reports or small series [36, 39–41]. Like SMARCA4-deficient carcinomas, they predominantly originate in the nasal cavity (up to 79% of cases) with a striking male predominance (83%) and a wide age range (mean: 50 years; range 10–82) [36, 39]. Most tumors present as large locally advanced masses (mean size: 7.4 cm) involving multiple sinonasal sites and invading the skull base. Only one third of patients remained disease-free after surgery and multimodal therapy, the remainder had either persistent local disease, local recurrence, distant metastases or have died [39]. In a recent systematic survival analysis review, the mean survival at 2 years was 55% [41].

Histologically, most TCSs demonstrated a predominance of undifferentiated primitive neuroepithelial elements with variable foci of neuropil-like matrix and rosettes. The epithelial component was mainly represented by immature clear cell (fetal-type) squamous islands but occasionally shows more mature-looking keratinizing epithelium. A glandular component with mucinous, intestinal-type or ciliated respiratory epithelium is frequently seen, but to a highly variable extent. The stromal mesenchymal component varies greatly as well from undifferentiated fibroblastic-type stromal elements (occasionally with periglandular condensation) to prominent rhabdomyoblastic features with strap cells. Less common stromal elements include foci of cartilage and bone.

The immunophenotype of the neoplastic cells in TCS corresponds to the line of differentiation of the diverse elements within a given tumor and is hence not specific, except for highlighting subtle components. Recently, our group have reported loss of SMARCA4 expression as the driver (sole molecular abnormality) in 82% of sinonasal TCSs by immunohistochemistry [39]. SMARCA4 loss was global across all tumor components in 68% and heterogeneous in 14% of cases. Cases with heterogeneous expression revealed SMARCA4 loss in the stromal elements but reduced or weak reactivity in the epithelial and the neuroepithelial components.

**Fig. 5:**
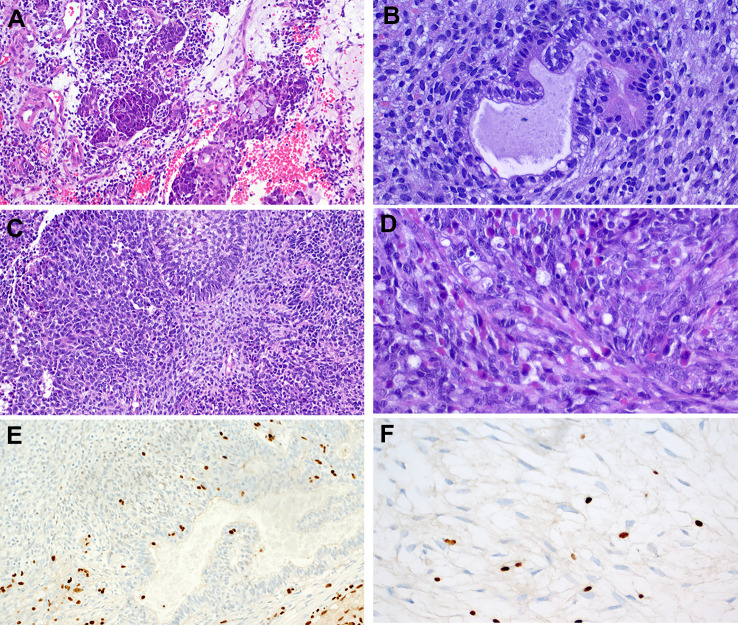
Representative examples of sinonasal teratocarcinosarcoma. **A** disordered admixture of mucinous glands, cohesive larger basaloid cell aggregates and primitive neuroectodermal-like stroma. **B** clear cell fetal-type tubules embedded within cellular primitive stroma. **C** clear cell fetal-type squamous islands (mid upper field) and scattered neuroepithelial rosettes (right) surrounded by highly cellular primitive stroma. **D** cellular spindled sarcomatoid stroma with numerous rhabdomyoblasts. **E** SMARCA4 loss is observed in stroma and in the glands. **F** mature-looking bland paucicellular stroma also shows SMARCA4 loss indicating it is neoplastic

**Fig. 6:**
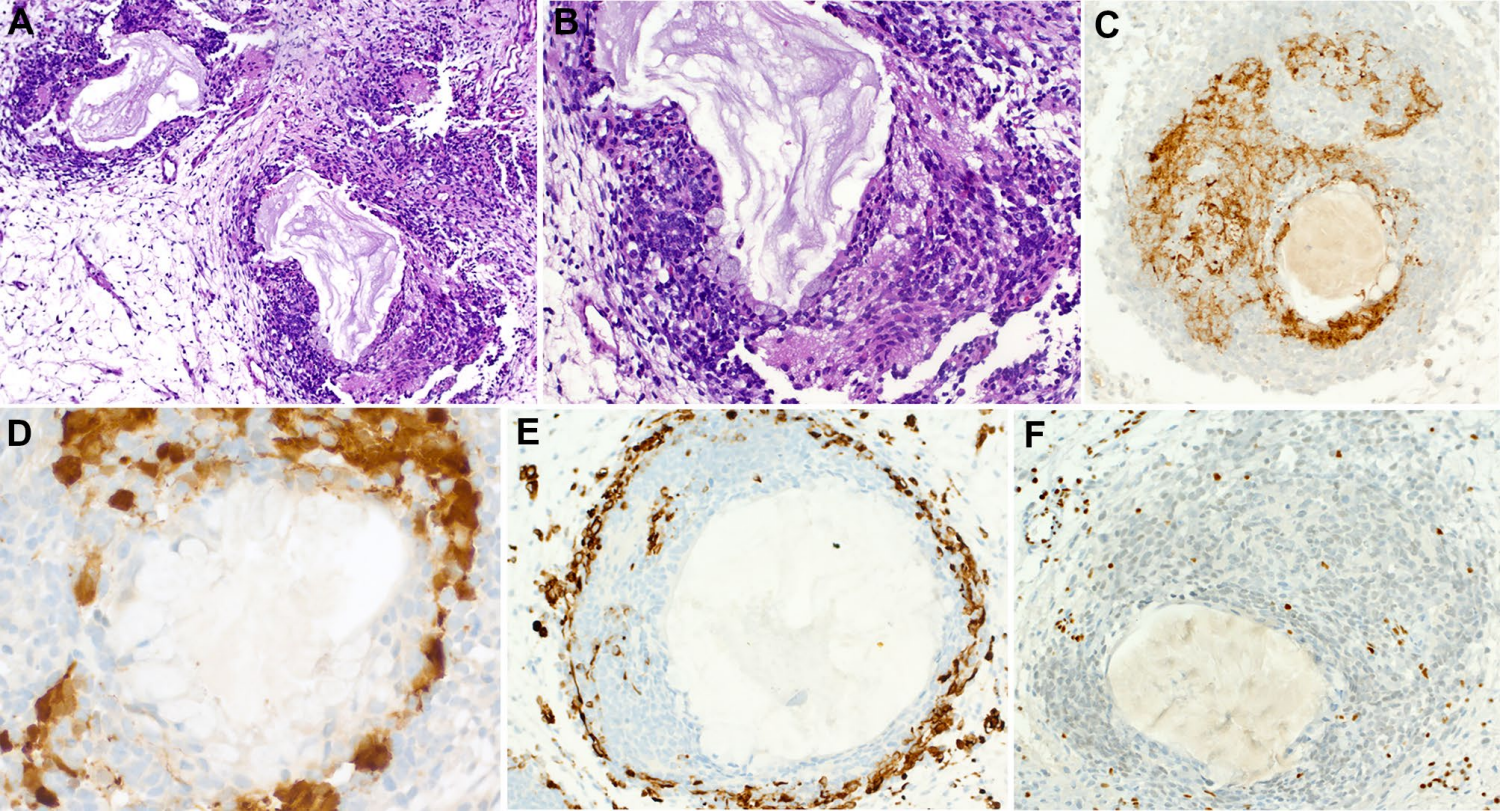
Further examples of sinonasal teratocarcinosarcoma. Some tumors display numerous organoid cellular “balls” (**A**) composed of centrally located dilated mucous glands surrounded by condensed primitive neuroectodermal-type stroma (**B**) highlighted by synaptophysin (**C**) and calretinin (**D**) and an outermost thin layer of primitive mesenchyme highlighted by desmin-immunostaining (**E**). **F** SMARCA4 loss is observed in all three cellular compartments

SMARCB1 expression is essentially intact, although a few cases may display variable low reactivity, but none showed complete loss of this protein [39]. Partial loss of SMARCA2 is a frequent finding in most tumors. Representative examples of the common and variant features of these tumors are depicted in Figs. 5 and 6.

### Differential Diagnosis and Conceptual Reappraisal of TCS

Remarkably, and due to their highly heterogeneous morphological patterns, sinonasal TCS may show significant overlap with almost any entity in the spectrum of poorly differentiated and undifferentiated malignancies, especially on limited biopsy material [14]. Accordingly, several entities such as olfactory neuroblastoma, poorly differentiated rhabdomyosarcoma, neuroendocrine carcinoma, high-grade non-intestinal adenocarcinoma, intestinal-type adenocarcinoma, squamous cell carcinoma, teratoma, odontogenic carcinosarcoma, and many others may be considered upon initial assessment of limited biopsy material. Predominance of one or the other component in different biopsies or in primary versus recurrent tumors should not be misinterpreted as discrepancy. SMARCA4-deficient carcinoma is the major consideration if SMARCA4 loss is identified in biopsies. Being unified by SMARCA4 loss, TCS and SMARCA4-deficient sinonasal carcinoma might be looked at as different morphotypes on the spectrum of same entity rather than two independent entities [32, 39]. However, in the experience of the author, rarity of carcinoma-like large cell components/ foci in majority of TCS argues to the contrary. It is the small blue cell (olfactory-like) pattern in the spectrum of SMARCA4-deficient sinonasal carcinoma that might be indistinguishable from TCS on limited biopsy material. In such a scenario, a descriptive diagnosis of “*SMARCA4-deficient sinonasal malignancy consistent with either carcinoma or TCS*” can be made prior to resection. Rarely, metastatic SMARCA4-deficient undifferentiated thoracopulmonary malignancies (indistinguishable from SMARCA4-deficient sinonasal carcinoma) might present in the head and neck sites, although very rare in the sinonasal areas, and should be thought of. Exploring the recent clinical history and review of current chest imaging are diagnostic [42].

The molecular pathogenesis of the small subset of SMARCA4-proficient TCS remains unknown. The role of *CTNNB1* mutations detected in some cases merits further investigation [43, 44].

### Therapeutic Implications of SWI/SNF Deficiency

Although no specific therapy exists for these aggressive neoplasms yet, current data suggests emerging roles for therapeutic opportunities targeting EZH2, CDK4/6 inhibitors and other potential targets in the treatment of these lethal diseases in clinical trials [45, 46]. Moreover, compiling evidence is accumulating that subsets of SWI/SNF-deficient malignancies may show enhanced response to immune therapies [47].

## Data Availability

Not applicable.
